# Positive end expiratory pressure in acute hypoxemic respiratory failure due to community acquired pneumonia: do we need a personalized approach?

**DOI:** 10.7717/peerj.4211

**Published:** 2018-01-30

**Authors:** Valentina Paolini, Paola Faverio, Stefano Aliberti, Grazia Messinesi, Giuseppe Foti, Oriol Sibila, Anna Monzani, Federica De Giacomi, Anna Stainer, Alberto Pesci

**Affiliations:** 1 Dipartimento Cardio-Toraco-Vascolare, Respiratory Unit, San Gerardo Hospital, ASST di Monza, University of Milan—Bicocca, Monza, Italy; 2 Department of Pathophysiology and Transplantation, Cardio-thoracic unit and Cystic Fibrosis Adult Center, Fondazione IRCCS Cà Granda Ospedale Maggiore Policlinico, University of Milan, Milano, Italy; 3 Department of Anesthesia and Intensive Care, San Gerardo Hospital, ASST-Monza, University of Milan— Bicocca, Monza, Italy; 4 Respiratory Department, Hospital de la Santa Creu i Sant Pau, Autonomous University of Barcelona (UAB), and Biomedical Research Institute Sant Pau (IIB Sant Pau), Barcelona, Spain

**Keywords:** Pneumonia, Respiratory failure, Continuous positive airway pressure, Positive end-expiratory pressure, Non-invasive ventilation

## Abstract

**Background:**

Acute respiratory failure (ARF) is a life-threatening complication in patients with community acquired pneumonia (CAP). The use of non-invasive ventilation is controversial. With this prospective, observational study we aimed to describe a protocol to assess whether a patient with moderate-to-severe hypoxemic ARF secondary to CAP benefits, in clinical and laboratoristic terms, from the application of a positive end expiratory pressure (PEEP) + oxygen vs oxygen alone.

**Methods:**

Patients who benefit from PEEP application (PEEP-responders) were defined as those with partial pressure of arterial oxygen to the fraction of inspired oxygen (PaO2/FiO2) increase >20% and/or reduction of respiratory distress during PEEP + oxygen therapy compared to oxygen therapy alone. Clinical characteristics and outcomes were compared between PEEP-responders and PEEP-non responders.

**Results:**

Out of 41 patients, 27 (66%) benefit from PEEP application (PEEP-responders), the best response was obtained with a PEEP of 10 cmH2O in 13 patients, 7.5 cmH2O in eight and 5 cmH2O in six. PEEP-responders were less likely to present comorbidities compared to PEEP-non responders. No differences between groups were found in regards to endotracheal intubation criteria fullfillment, intensive care unit admission and in-hospital mortality, while PEEP-responders had a shorter length of hospital stay.

**Discussion:**

The application of a protocol to evaluate PEEP responsiveness might be useful in patients with moderate-to-severe hypoxemic ARF due to CAP in order to personalize and maximize the effectiveness of therapy, and prevent the inappropriate PEEP use. PEEP responsiveness does not seem to be associated with better outcomes, with the exception of a shorter length of hospital stay.

## Introduction

Acute respiratory failure (ARF) is a common life-threatening condition caused by the inability of the respiratory system to maintain normal levels of oxygen and/or carbon dioxide in the blood (Williams et al., 2012). Oxygen supplementation is the mainstay of treatment for ARF, however, in more severe cases invasive mechanical ventilation (IMV) may be required. IMV requires an intensive care unit (ICU) to be performed and is subdued to high complications rates (Williams et al., 2012). Given these limitations, an increasing number of patients with moderate to severe ARF receive a non-invasive ventilation (NIV) trial before undergoing endotracheal intubation (ETI). The term NIV comprehends a number of different ventilatory modes, including both continuous positive airway pressure (CPAP) and bilevel positive airway pressure (BiPAP), and different patient-ventilator interfaces, such as helmet and face or oronasal masks.

The efficacy of NIV in patients with ARF mainly depends on the severity and the cause of the ARF itself (Nicolini et al., 2015; Ferrer, Cosentini & Nava, 2012; Ferrer & Torres, 2015). The use of NIV should be limited to patients with less severe ARF (partial pressure of arterial oxygen to the fraction of inspired oxygen [PaO2/FiO2] at presentation >150, or PaO2/FiO2 after 1 h from NIV onset >175) with close monitoring and management by experienced personnel in a high-dependency unit in order to detect early signs and symptoms of NIV failure (Nicolini et al., 2015). NIV is also associated to a good response in ARF patients with preexisting cardiac or respiratory diseases, such as pulmonary edema and chronic obstructive pulmonary disease (COPD) exacerbations (Ferrer & Torres, 2015).

The use of NIV in patients with pneumonia is controversial, with the exception of immunosuppressed patients, in whom NIV may decrease the need for IMV and improve the poor outcome associated with ETI and ICU stay (Ferrer, Cosentini & Nava, 2012). Recent evidence has shown that patients with moderate-to-severe hypoxemic ARF due to pneumonia treated with CPAP delivered by helmet had a more rapid improvement of arterial oxygenation and a lower risk of meeting ETI criteria when compared to the standard oxygen therapy (Cosentini et al., 2010; Brambilla et al., 2014).

To date, the heterogeneity of the populations analyzed in the available studies has shown that not all patients benefit from the application of a Positive End-Expiratory Pressure (PEEP) (Ferrer & Torres, 2015; Luo et al., 2016).

Patients may not respond to CPAP application for several reasons, including intolerance to the interface or pressure applied and development of hemodynamic instability (Ferrer & Torres, 2015; Luo et al., 2016). The application of a protocol to readily distinguish patients that benefit from PEEP application (PEEP-responders) from those that do not (PEEP-non responders) could help to prevent ETI delay and inappropriate NIV use, to individualize ventilatory and oxygenation support optimizing PEEP use.

Primary aim of the present study is to describe a protocol that assesses whether a patient with moderate-to-severe hypoxemic ARF due to CAP benefits, in clinical and laboratoristic terms, from the application of a PEEP + oxygen therapy through helmet-CPAP *vs* oxygen therapy alone. Secondary aim is to evaluate the prevalence, clinical characteristics and outcomes of PEEP-responder in comparison to PEEP-non-responder patients.

## Matherials & Methods

### Study design

This was a single-center prospective cohort study of patients admitted with a diagnosis of hypoxiemic ARF due to CAP who underwent a CPAP trial in the respiratory high dependency unit (HDU), between January 2013 and August 2016. The institutional review board of the hospital approved the study (#57/January 2013) and patients signed an informed consent form.

### Study population

Patients were included in the study if they presented all the following characteristics:

- age ≥ 18 years;
- diagnosis of CAP (healthcare associated pneumonia was included in the CAP definition)
- acute hypoxiemic respiratory failure (PaCO_2_ ≤ 45 mmHg) with a PaO_2_/FiO_2_ ≤ 200 during supplementation of oxygen with Venturi mask FiO2 35%, 50% or non-rebreathing mask at a flow rate of 15 L/min for at least 20 consecutive minutes with a target SpO2 ≥90%.

Patients with at least one among the following were excluded from the study:

• diagnosis of hospital-acquired pneumonia;
• concomitant diagnosis of undrained pneumothorax or pulmonary embolism;
• chronic CPAP use to correct obstructive sleep apnea syndrome (OSAS) if the same mode and setting were maintained during hospitalization for pneumonia;
• unstable hemodynamic conditions;
• severe central neurological disturbance (e.g., comatose state);
• inability to protect respiratory airways;
• inability to fit the interface (e.g., facial trauma or burns);
• presence of open wound (skull, chest, abdomen);
• severe gastrointestinal bleeding;
• uncooperative patient;
• active pregnancy;
• respiratory arrest and ETI need;
• Concomitant diagnoses, such as COPD exacerbations and pulmonary edema, that could have been the primary cause of ARF instead of pneumonia according to the medical team in charge of the patient.

### Data collection

Demographics, comorbidities, laboratory, microbiological and radiological tests performed upon diagnosis of pneumonia, severity of the disease and antibiotic therapy used upon CPAP-trial were collected for all the enrolled patients. Patients were followed up till hospital discharge. Length of CPAP application (in PEEP-responder patients) and adverse events as well as intolerance related to CPAP application were also registered.

### Modalities of CPAP application

CPAP was delivered in all patients enrolled in the study through a high-flow generator (90–140 L/min; VitalSigns, Inc., San Antonio, TX, USA; StarMed, Mirandola, Italy) using a helmet interface (StarMed, Mirandola, Italy) with a PEEP water valve (Harol, Milan, Italy). To maintain stable FiO2 values at different PEEP levels, oxygen flow was modified in accordance with the tables provided by the manufacturer. FiO2 was initially titrated with a Venturi mask or non-rebreathing mask at a flow rate of 15 L/min to maintain a pulse oximetry ≥90% for at least 20 min. CPAP was then applied with the same FiO2 setting used with oxygen supplementation alone.

### Study outcomes

The following primary end-points were evaluated:

- *ETI criteria fullfillment:* presence of at least one of the following major criteria or at least two of the following minor criteria.
  Major criteria:
    - cardiac arrest;
    - hemodynamic instability (SBP <90 mmHg, despite adequate fluid resuscitation);
    - inability to protect the airways.
  Minor criteria:
    - PaCO2 increase ≥ 45 mmHg;
    - significant worsening or onset of respiratory distress;
    - neurological impairment (agitation requiring patient’s sedation);
    - PaO2/FiO2 decrease >20% compared to baseline.
- Admission to ICU.
- *In-hospital mortality:* defined as death by any cause occurred during hospitalization.

The following were considered as secondary outcomes:

- *Length of hospital stay (LOS)*: calculated as the number of days from the date of admission to the date of discharge;
- *Switch from CPAP to BiPAP ventilation:* adding of a “Pression Support” (PS) in case of onset or persistence of respiratory distress and/or PaCO2 increase ≥45 mmHg.

### Trial planning and evaluation of response to FiO2 and PEEP application

If a patient had a diagnosis of pneumonia, fullfilled all inclusion and exclusion criteria and signed informed consent, he/she was eligible to participate in the study and to perform the CPAP trial.

**Figure 1:**
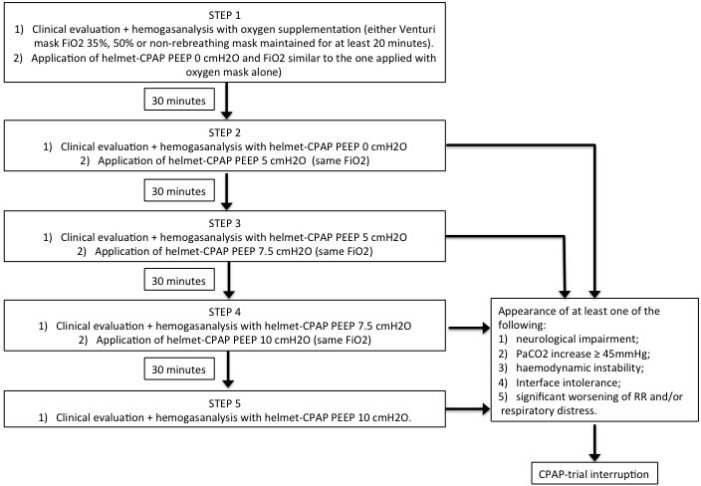
Description of the CPAP-trial used to differentiate PEEP responder patients from PEEP-non responder. PEEP, Positive End-Expiratory Pressure; CPAP, continuous positive airway pressure; FiO2, fraction of inspired oxygen; RR, respiratory rate.

The steps of the CPAP trial are summarized in Fig. 1. During the clinical evaluation performed in each step we measured vital signs, including blood pressure (BP), heart rate (HR), and respiratory rate (RR), and evaluated the presence of respiratory distress, defined as accessory muscles use and/or chest-abdominal dyssynchrony and paradoxical respiration. Helmet-CPAP tolerance and level of consciousness (through patients’ response to simple verbal commands if alert, or through eye opening, verbal and motor response if not alert) were also assessed.

**Figure 2:**
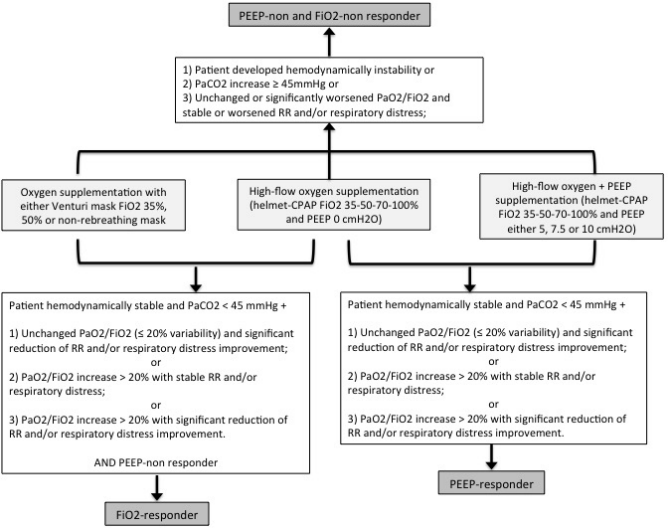
Definitions of PEEP-responder, FiO2 responder, PEEP-non and FiO2-non responder. PEEP, Positive End-Expiratory Pressure; FiO2, fraction of inspired oxygen; RR, respiratory rate; CPAP, continuous positive airway pressure. Significant changes in clinical or arterial blood gas parameters are intended as an improvement or worsening of 20% compared to baseline.

The definitions of PEEP-responder, FiO2-responder and PEEP-non and FiO2-non responder are presented in Fig. 2. FiO2-responsiveness was evaluated comparing clinical and arterial blood gas parameters during oxygen supplementation with Venturi mask and helmet-CPAP PEEP 0 cmH2O, maintaining the same FiO2. A FiO2-responder is defined as a subject with clinical and/or arterial blood gases improvement with helmet-CPAP PEEP 0 cmH2O compared to Venturi mask, maintaining the same FiO2. PEEP-responsiveness was evaluated comparing clinical and arterial blood gas parameters during oxygen supplementation with helmet-CPAP PEEP 0 cmH2O and other PEEP levels (5, 7.5 and 10, respectively), maintaining the same FiO2. A PEEP-responder is defined as a subject with clinical and/or arterial blood gases improvement with helmet-CPAP PEEP 5, 7.5 or 10 cmH2O compared to helmet-CPAP PEEP 0 cmH2O, maintaining the same FiO2.

### Statistical analysis

Data were analyzed using SPSS 21.0 for MAC OS (SPSS Inc., Chicago, IL, USA). Baseline characteristics of the study population, biomarkers levels, radiological features on admission, and outcomes were considered for statistical analysis. Continuous variables are expressed as median (interquartile range -IQR-25th–75th percentile) and compared using Wilcoxon–Mann–Whitney U two-sample test. Categorical data are expressed as frequencies and percentages and compared using the chi-square or Fisher exact test, where appropriate. All tests were 2-tailed and a *p*-value <0.05 was considered statistically significant.

## Results

### CPAP-trial results

A total of 41 patients (median age 74 years, 71% males) were enrolled in the study.

Study population was divided according to the response to the CPAP-trial in two main groups: PEEP-responders (27 patients, 66%) and PEEP-non responders (14 patients, 34%). In the latter group, the reasons for PEEP-non responsiveness were as follows: 11 patients showed unchanged or worsened clinical and arterial blood gas parameters (in terms of oxygenation) during CPAP application, two patients (both with a history of COPD) developed hypercapnia after CPAP application, and one patient experienced intolerance to the interface.

PEEP-non responders were further subgrouped in FiO2-responders (11 patients—27% of the entire study population) and PEEP-non and FiO2-non responders (three patients—7% of the entire study population).

During the CPAP trial vital signs and arterial blood gas parameters were evaluated with oxygen mask and with Helmet-CPAP at different PEEP levels (0, 5, 7.5 and 10 cmH2O), Table 1.

**Table 1:** Vital signs and arterial blood gas parameters during CPAP-trial.

	Oxygen only	PEEP 0 cmH2O	PEEP 5 cmH2O	PEEP 7.5 cmH2O	PEEP 10 cmH2O
PaO2/FiO2, median [IQR]					
Entire population	149 [131–178]	202 [147–268]	223 [172–315]	230 [168–296]	272 [167–354]
PEEP-responders	151 [136–173]	202 [126–257]	234 [189–328]	255 [195–302]	280 [209–361]
PEEP-non responders	145 [128–196]	227 [166–282]	210 [152–291]	208 [134–320]	175 [129–294]
RR, median [IQR]					
Entire population	26 [20–31]	24 [20–30]	24 [20–29]	24 [21–29]	26 [21–29]
PEEP-responders	30 [20–33]	26 [24–30]	24 [22–30]	24 [22–28]	26 [23–29]
PEEP-non responders	20 [20–30]	23 [16–31]	23 [20–29]	25 [20–31]	25 [20–30]
PaCO2 (mmHg), median [IQR]					
Entire population	37 [33–39]	38 [33–40]	37 [33–41]	37 [32–39]	38 [34–40]
PEEP-responders	37 [33–39]	36 [29–39]	36 [33–39]	36 [32–38]	37 [32–40]
PEEP-non responders	38 [36–40]	40 [37–41]	40 [36–43]	39 [33–43]	40 [37–43]
HR, median [IQR]					
Entire population	86 [80–100]	81 [79–94]	80 [73–92]	87 [74–95]	86 [74–97]
PEEP-responders	80 [78–97]	80 [78–92]	80 [70–92]	81 [70–94]	82 [70–90]
PEEP-non responders	90 [80–106]	86 [79–97]	82 [75–98]	91 [76–98]	95 [78–103]
MAP, median [IQR]					
Entire population	87 [80–93]	90 [81–100]	93 [83–99]	92 [86–106]	94 [88–104]
PEEP-responders	87 [81–94]	90 [81–100]	92 [83–97]	90 [83–100]	94 [85–104]
PEEP-non responders	84 [80–93]	86 [81–100]	93 [86–105]	103 [89–113]	98 [90–109]
Lactate (mMol/L), median [IQR]					
Entire population	1.7 [1.1–1.9]	1.4 [1–1.9]	1.3 [1–2]	1.2 [1–2]	1.2 [0.9–1.6]
PEEP-responders	1.7 [1.1–2.1]	1.5 [1–1.9]	1.5 [0.9–2]	1 [1–2]	1.2 [1–1.5]
PEEP-non responders	1.3 [0.9–3.2]	1.3 [0.7–1.9]	1.2 [0.8–1.8]	1.3 [1–1.3]	1.2 [0.7–2.1]

**Notes.**

PEEP Positive End Expiratory Pressure PaO2/FiO2 partial pressure of arterial oxygen to the fraction of inspired oxygen RR respiratory rate MAP mean arterial pressure HR heart rate

Among the 27 PEEP-responder patients, the best response was obtained with a PEEP level of 10 cmH2O in 13 patients, 7.5 cmH2O in eight patients, and 5 cmH2O in six patients.

### Characteristics of study population

Demographics, comorbidities, laboratory tests and radiological features of the study population are summarized in Table 2. No statistically significant differences were found between groups in regards to demographics, laboratories and radiological features of pneumonia upon execution of CPAP-trial. In regards to comorbidities, chronic liver disease and chronic renal failure were more common in PEEP-non responder patients compared to PEEP-responders.

**Table 2:** Demographic and clinical characteristics of the study population.

	Entire population (*N* = 41)	PEEP-responders (*N* = 27)	PEEP-non responders (*N* = 14)	*p*-value
**Demographics, n. (%)**				
Age (years), median [IQR]	74 [59–80]	68 [45–79]	78 [68–82]	0.055
Males	29 (71)	18 (67)	11 (79)	0.49
BMI (kg/m^2^), median [IQR]	25 [22.8–27.9]	25 [22.9–28.3]	25 [20.8–26.5]	0.61
Current or prior smokers	27 (66)	16 (59)	11 (79)	0.31
**Comorbidities, n. (%)**				
Coronary artery disease	6 (15)	4 (15)	2 (14)	1
Valvular heart disease	2 (5)	2 (7)	0	0.54
Hypertension	18 (44)	14 (52)	4 (29)	0.2
Chronic arrhythmia	10 (24)	7 (26)	3 (21)	1
COPD	12 (29)	5 (19)	7 (50)	0.068
Pulmonary emphysema	6 (15)	2 (7)	4 (29)	0.16
Long-term oxygen therapy	4 (10)	2 (7)	2 (14)	0.6
Chronic renal failure	3 (7)	0	3 (21)	**0.03**
Chronic liver disease	6 (15)	1 (4)	5 (36)	**0.01**
Active solid cancer	2 (5)	0	2 (14)	0.11
Hematologic malignancy	7 (17)	4 (15)	3 (21)	0.66
Cerebrovascular disease	2 (5)	1 (4)	1 (7)	1
Dementia	1 (2)	1 (4)	0	1
Diabetes mellitus	7 (17)	6 (22)	1 (7)	0.39
Autoimmune disease	3 (7)	2 (7)	1 (7)	1
Immunosuppression	4 (10)	4 (15)	0	0.28
**Laboratories, median [IQR]**	****	****	****	****
WBC (×10^3^/mL)	12.1 [7.8–16.5]	10.5 [7.8–14.2]	14.1 [7.8–21]	0.24
Hemoglobin (g/dL)	12.3 [11–13.3]	12.3 [11–13.6]	12.4 [10.5–13.2]	0.86
Platelets (×10^3^/mL)	197 [137–265]	193 [132–263]	208 [142–293]	0.73
C-reactive protein (mg/dL)	19 [9.3–28.5]	15.7 [6.3–25]	25.3 [17–31.3]	0.07
**Radiological features, n. (%)**				
Left upper lobe involvement	9 (22)	6 (22)	3 (21)	1
Left lower lobe involvement	17 (42)	12 (44)	5 (36)	0.74
Right upper lobe involvement	15 (37)	10 (37)	5 (36)	1
Middle lobe involvement	6 (15)	3 (11)	3 (21)	0.39
Right lower lobe involvement	21 (51)	12 (44)	9 (64)	0.33
Interstitial pneumonia	3 (7)	3 (11)	0	0.54
Unilobar involvement	18 (44)	14 (52)	4 (29)	0.2
Multilobar involvement	23 (56)	13 (48)	10 (71)	0.2

**Notes.**

PEEP Positive End Expiratory Pressure IQR interquartile range 25th–75th percentile BMI body mass index COPD chronic obstructive pulmonary disease WBC white blood cells

Significant *p*-values (*p* < 0.05) are in bold.

Severity of the disease upon execution of CPAP-trial is summarized in Table 3. No differences were found between groups both in regards to severity score (Sequential Organ Failure Assessment (SOFA) score) and neurological and hemodynamic parameters.

**Table 3:** Severity of the disease upon CPAP-trial execution.

	Entire population (*N* = 41)	PEEP-responders (*N* = 27)	PEEP-non responders (*N* = 14)	*p*-value
**Severity of the disease, n. (%)**				
SOFA score, median [IQR]	4 [3–4]	4 [3–4]	4 [3–4]	0.93
Alteration of mental status	2 (5)	1 (4)	1 (7)	1
Vasopressors use	3 (7)	3 (11)	1 (7)	0.54
Diuresis < 0.5 ml/kg/h	4 (10)	1 (4)	3 (21)	0.09
Hypotension requiring fluid	7 (17)	4 (15)	3 (21)	0.66
resuscitation				

Etiological diagnosis of CAP was reached in 16 patients (34%) and the following microorganisms were the most commonly isolated: *S. pneumoniae* (six patients), *L. pneumophila* (five patients), *methicillin-resistant S. aureus* (one patient), *P . aeruginosa* (one patient), *M. catarrhalis* (one patient), *P. Jiroveci* (one patient), influenza B virus (one patient), with no statistical significant difference between the two groups.

Antibiotic therapy was chosen in accordance with international guidelines and did not differ between groups (Woodhead et al., 2011).

### Helmet-CPAP tolerance and adverse events

Thirty-eight patients (93%) completed the CPAP trial. The reasons for CPAP-trial interruption were PaCO2 increase ≥45 mmHg in two cases and intolerance to the interface in one case. In PEEP-non responder patients CPAP was immediately discontinued. In the PEEP-responder group the median [IQR] length of CPAP treatment was 5 [3–7] days. In this group of patients, helmet-CPAP was applied three cycles per day (morning, afternoon and night) and each cycle consisted of at least a 3-hour application. If clinical parameters and gas exchange allowed it, helmet-CPAP was interrupted for oral feeding and personal hygiene. Helmet-CPAP usefulness was assessed on a daily basis through clinical and hemogasanalytic evaluation; once respiratory failure and distress were solved it was discontinued. None of the PEEP-responder patients developed discomfort, due to intolerance to the interface and/or pressure applied, or pressure ulcers.

Cardiovascular complications developed in three cases (7%) during hospital stay, but not during the CPAP-trial: two cases of new onset supraventricular tachyarrhythmia (one PEEP-responder and one PEEP-non responder patient, respectively, *p*-value 1), and one case of acute coronary syndrome in a PEEP-responder patient.

### Study outcomes

Among the primary study outcomes, ETI criteria were fullfilled by one patient in the PEEP-non responder group (simultaneous achievement of two minor criteria: onset of respiratory distress and PaO2/FiO2 decrease >20% compared to baseline), regardless of the presence of a do-not-intubate-do-not-resuscitate (DNI-DNR) order. One patient (PEEP-responder) required ICU admission (one day after performing the CPAP-trial). Three patients (7%) died during hospitalization with no statistically significant differences between groups (one *vs* two patients in the PEEP-responder and PEEP-non responder group, respectively, *p*-value 0.54).

Among the secondary end-points, the median LOS showed a statistically significant difference between study groups (13 days [IQR 9-18] in the PEEP-responder *vs* 18 days [IQR 14-24] in the PEEP-non responder group, *p*-value 0.048). One PEEP-non responder patient, who developed hypercapnia during the CPAP-trial, was switched from CPAP to BiPAP ventilation.

## Discussion

In the present study we described a protocol to perform a rapid and repeatable CPAP trial that allowed to select patients with hypoxemic ARF due to CAP who benefit from PEEP application (PEEP-responders) and to determine the optimal PEEP level. No differences were found between PEEP-responder and PEEP-non responder patients in regards to the main clinical, laboratoristic and radiological features, with the exception of a higher burden of comorbidities in PEEP-non responders. Failure to respond to PEEP application was not associated with worse outcomes, with the exception of a significantly longer length of hospital stay in PEEP-non responder patients.

In our cohort, 34% of patients with hypoxemic ARF due to CAP did not benefit from PEEP application, thus, our protocol allowed a personalized approach and a limitation to the inappropriate use of PEEP. Furthermore, among PEEP-non responders, our protocol allowed the identification of patients that benefit from the application of high flow oxygen (FiO2-responders), who might be the best candidates for high-flow nasal cannula trials.

There are also limited data on what is the optimal PEEP level to apply in patients with ARF due to pneumonia (Cosentini et al., 2010; Brambilla et al., 2014). In the studies conducted so far, a PEEP level of 10 cmH2O has been applied to all patients randomized in the NIV arm (Cosentini et al., 2010; Brambilla et al., 2014). In our cohort, more than half of the PEEP-responder patients showed a better clinical and hemogasanalytic response with a PEEP value lower than 10 cmH2O. This result suggests that there is not a standard PEEP applicable to all patients, but each subject requires an individualized value; however, the factors that determine the response to a higher or lower PEEP level in patients with pneumonia still need to be identified.

The CPAP trial we proposed also allowed, for the first time, to compare the characteristics of PEEP-responder *vs* PEEP-non responder patients. We did not find any difference between the two groups, with the exception of a lower burden of comorbidities in PEEP- responder patients. In particular, no differences were found in regards to radiological features and severity of the disease.

In our study CPAP was applied through helmet instead of face or oral-nasal mask. This interface was well tolerated and showed few side effects (only one patient discontinued the trial due to helmet intolerance). Our results are supported by other similar observations by *Brambilla et al. and Patel and colleagues*, who confirmed the good tolerance to helmet in patients with ARF due to pneumonia and ARDS (Acute Respiratory Distress Syndrome), respectively (Brambilla et al., 2014; Patel et al., 2016).

In our cohort, we did not find statistically significant differences between PEEP- responders and PEEP-non responders in regard to the primary outcomes, although patients in the latter group were older, more likely to receive a DNR-DNI order and with more comorbidities. Therefore, the failure to respond to PEEP application alone does not seem to be a poor prognostic factor in patients with hypoxemic ARF due to CAP.

Among the secondary outcomes, we observed a longer duration of hospital stay in PEEP-non responders. A possible explanation for this finding may lay, as already noted, in the older age and the greater burden of comorbidities of these patients.

Finally, in our cohort we found that three patients, with no statistically significant difference between PEEP- responders and PEEP-non responders, developed cardiovascular complications during hospital stay, but not during CPAP trial. The increased incidence of cardiovascular events in patients with pneumonia, up to a quarter of those admitted to the hospital, has been extensively described in a series of papers by Corrales-Medina et al. (2012).

To the best of our knowledge, this is the first study to describe a protocol to perform a rapid and repeatable CPAP trial that allows to identify PEEP-responder patients and the optimal PEEP level, however, a few limitations have to be highlighted. First of all, although our study was conducted prospectively, the small number of patients recruited in a single center and the use of helmet-CPAP as interface instead of face masks limit the generalizability of the data and the possibility to perform further analyses on FiO2-responder patients and different PEEP levels. Secondly, having carried out the trial only in patients hospitalized in a respiratory HDU, a selection bias must be considered: patients with low performance status, advanced age and multiple comorbidities were often not considered eligible for admission in the respiratory HDU and, thus, excluded from the study. Conversely, patients candidates to invasive maneuvers that showed a rapid clinical deterioration at presentation may have been directly admitted to ICU for the incipient risk of ETI. Thirdly, our cohort of patients was not homogeneous with regards to the temporal phase of pneumonia: the CPAP trial was performed both in patients at early stages and in post-acute stages of the disease (e.g., during weaning from IMV). Finally, we did not include in the study a control group, because the application of the CPAP trial is standard-of-care for all patients with moderate to severe hypoxemic ARF due to pneumonia admitted to our respiratory HDU.

Future studies should aim to the external validation of the CPAP trial and to identify the predictors of response to PEEP application.

## Conclusions

In conclusion, the application of the CPAP trial we proposed might be useful in patients with moderate-to-severe hypoxemic ARF due to CAP in order to personalize and maximize the effectiveness of therapy, and prevent the inappropriate use of PEEP. PEEP responsiveness was not associated with better outcomes, with the exception of a shorter LOS.

##  Supplemental Information

10.7717/peerj.4211/supp-1 Supplemental Information 1 Raw data—PEEP responders

## Additional Information and Declarations

### Data Availability

The following information was supplied regarding data availability: The raw data is provided as a Supplemental File.
